# Editorial: Pharmacology of infectious diseases: world tuberculosis day 2022

**DOI:** 10.3389/fphar.2023.1233347

**Published:** 2023-06-13

**Authors:** Shashikant Srivastava, Johannes W. Alffenaar, Sebastian G. Wicha

**Affiliations:** ^1^ Department of Medicine, School of Medicine, University of Texas at Tyler, Tyler, TX, United States; ^2^ Department of Cellular and Molecular Biology, University of Texas Health Science Center at Tyler, Tyler, TX, United States; ^3^ Center for Biomedical Research, University of Texas Health Science Centre at Tyler, Tyler, TX, United States; ^4^ Sydney Institute for Infectious Diseases, The University of Sydney, Sydney, NSW, Australia; ^5^ Faculty of Medicine and Health, School of Pharmacy, The University of Sydney, Sydney, NSW, Australia; ^6^ Westmead Hospital, Sydney, NSW, Australia; ^7^ Department of Clinical Pharmacy, Institute of Pharmacy, University of Hamburg, Hamburg, Germany

**Keywords:** pharmacology, infectious diseases, tuberculosis, drug discovery, modeling

Robert Koch discovered *Mycobacterium tuberculosis* (*Mtb*), the causative agent of tuberculosis (TB), in 1882 (Koch, 1882). Significant progress has been made since then to treat TB and now we have treatment regimens with a four-month duration for drug-susceptible TB (Gillespie et al., 2014) and the possibility that the therapy duration can further be shortened (Gumbo et al., 2022). However, the emergence of drug resistance during therapy remains a major challenge and continuous efforts are needed to develop new drugs, regimens, and biomarkers for therapy outcomes. The series of articles in this Research Topic of “Frontiers in Pharmacology” provides an insight into the pharmacokinetics/pharmacodynamics (PK/PD) of anti-tuberculosis drugs, various mathematical and computer-aided simulation models on the PK/PD as well methods to use Mycobacteria Growth Indicator Tube (MGIT) derived time-to-positive (TTP) as a biomarker for therapeutic outcome.

The first review article, by Alffenaar et al. discusses *in vitro*, *in vivo,* and human studies, the factors to consider in a study design, the importance of uniform reporting including microbiological and clinical outcome assessments, and modeling approaches to link the PK/PD and other output from these studies. Regarding the *in vitro* studies examining the efficacy of drugs against *Mtb*, the authors discussed the importance of determining the MIC using various methods, time-kill kinetic studies at static concentrations, and how a dynamic hollow fiber system of tuberculosis (HFS-TB) can be used not only to determine the drug exposure for *Mtb* kill and resistance suppression but also to investigate different metabolic populations of *Mtb*. Regarding the animal studies, Alffenaar et al. discussed the effect of routes of infection, PK in blood versus at the site of infection, PD read-outs (CFU and TTP), biomarkers and use of the *in vivo* systems to evaluate the differences in treatment in terms of sterilization on infection, relapse, and death. The review also discusses the human PKPD with a focus on early bactericidal activity (EBA) studies and proposed the inclusion of PK and therapeutic drug monitoring (TDM) in clinical trials. An overview of the *in silico* modeling and computer-aided clinical trial simulation to link *in vitro*, *in vivo,* and human studies were also included to discuss how the modeling approach can bridge the translation of pre-clinical findings to clinical settings.

The second review article in the series, by Stadler et al. is on clofazimine that was introduced in the clinics due to the reported *in vitro* anti-TB activity but without the PK and safety evaluation. Interestingly, while clofazimine lacks the EBA, is synergistic with many first- and second-line drugs. This review summarizes the clinical studies on the safety and efficacy outcomes of clofazimine-containing regimens as well as a comprehensive list of recently completed and ongoing clinical trials to investigate the efficacy of clofazimine against both drug-susceptible and drug-resistant forms of TB. Stadler et al. literature review also provides a rationale for developing novel clofazimine analogs with improved antimycobacterial activity as well as better PK and tolerability profile, to add to the armament of anti-TB drugs.

Linezolid is one of the core drugs now included in the multidrug-resistant (MDR)-TB treatment regimen, including the BPaLM regimen recommended by the World_Health Organizaton (2022). Zhang et al. performed a prospective multi-center cohort study including 168 participants with MDR-TB for clinical validation of the linezolid exposure target identified in the HFS-TB (Srivastava et al., 2017). The authors used a population PK model for linezolid and used classification and regression tree analyses (CART) to identify the ratio of 0–24 h area under the concentration-time curve (AUC_0-24_) to the minimal inhibitory concentration (MIC) associated with successful treatment outcome. The authors analyzed a total of 1,008 linezolid plasma concentrations using a two-compartment model with first-order absorption and elimination to identify AUC_0–24_/MIC of >125 as the threshold for successful treatment outcome, close to the threshold identified in the HFS-TB as an AUC_0–24_/MIC of 119 (Srivastava et al., 2017). The time to culture conversion in patients with AUC_0-24_/MIC >125 was also similar to the HFS-TB predicted 2-month threshold. The authors concluded that with the standard 600 mg once daily dose of linezolid, the probability of target attainment was 100% up to the MIC ≤0.25 mg/L. Thus, Zhang et al. study provides crucial clinical evidence that preclinical models, such as HFS-TB, combined with mathematical modeling and clinical trial simulations can accurately predict clinical outcomes.

The second study on linezolid by Resendiz-Galvan et al. compared the therapeutic outcomes and adverse events between linezolid 600 mg versus 300 mg daily dose in patients with MDR-TB treated at a tertiary care hospital in Mumbai, India. Intensive or sparse blood sampling was performed for the PK analysis in 183 study participants. The authors concluded that the likelihood of failing to achieve the optimal exposure target with linezolid 300 mg once daily dose was 21%, whereas the probability of target attainment (PTA) of 600 mg daily dose was >90%. However, there was a higher likelihood of exceeding toxicity thresholds with a 600 mg dose (31%) versus the 300 mg daily dose (9.6%). The findings of Resendiz-Galvan et al. also highlight another aspect of personalized medicine, i.e., therapeutic drug monitoring (TDM) to individualize linezolid dosing to improve the treatment efficacy while balancing the risk of drug-induced toxicity for the treatment of MDR-TB (Nahid et al., 2019; Alffenaar et al., 2022).

Bedaquiline is included in the treatment regimen for MDR-TB. However, measurement of bedaquiline plasma concentrations could be tricky, thus information on drug exposure to associate with treatment outcome is limited. The study by Shao et al. performed population PK modeling, using a three-compartment model, to evaluate the WHO-recommended 400 mg once daily dose for 14 days followed by 200 mg three times a week after 2–4 weeks of treatment. The model was developed using 1,205 bedaquiline plasma concentrations from 55 patients and showed a wide concentration range between 0.04 mg/L to 5.96 mg/L. The model validation cohort included 159 patients. Patients on bedaquiline treatment achieving an AUC_0-24_/MIC >175.5 were predicted to have a higher probability of culture conversion after two-months of therapy. The study also reported that in 13 patients bedaquiline was either discontinued or a dose reduction was required due to moderate and serious QT prolongation.

To exert the antimicrobial effect, penetration of the drugs at the site of infection is an important factor when selecting the drugs in a combination regimen (Dheda et al., 2018). TB meningitis is difficult to treat as many of the drugs do not penetrate the central nervous system. Maranchick et al. evaluated the cerebrospinal fluid (CSF) concentrations of fluoroquinolones and carbapenems in patients with TB meningitis. The authors evaluated levofloxacin, moxifloxacin, ofloxacin, imipenem, and meropenem concentrations in 22 patients treated for TB meningitis, among them only eight were confirmed cases, highlighting the difficulty of establishing a diagnosis in central nervous system TB. The levofloxacin CSF/serum ratio at 2–6 h sampling time points was 0.41–0.63, respectively. For moxifloxacin, the adjusted CSF/serum ratios at 2–6 h sampling time points were 0.44–0.62, respectively. While Maranchick et al. study evaluated only two time points, a previous study with intensive PK sampling reported that moxifloxacin C_max_ in the CSF was observed after 4hrs of drug administration, and the mean plasma to CSF AUC ratio for total concentrations was 0.82 (range 0.70–0.94). Therefore, the observed difference between the two studies could be the timepoint of sampling. This also highlights the importance of performing full PK instead of sparse sampling for drugs, where toxicity could be a concern, to calculate the actual drug exposure and dose adjustments as needed. Regarding the carbapenems, among the 76 CSF samples, the authors reported that drug concentration was below the limit of detection in 79% of samples. In summary, the findings of Maranchick et al. reinforce the use of fluoroquinolones in TB meningitis, but questions carbapenem’s utility in the treatment of TB meningitis.

Continuing the search for drugs with penetration at disease-specific anatomic sites, the study by Deshpande et al. used the HFS-TB to determine the potential role of minocycline in the treatment of TB. The same study also tested several combinations of minocycline with tedizolid, moxifloxacin, and rifampin mimicking intrapulmonary PK of each drug. In addition, Deshpande et al. explored the possibility of an intermittent regimen by combining minocycline with tedizolid. Notably, the authors determined the intra-bacterial minocycline PK as a potential explanation for the effectiveness of the proposed once-a-week regimen. The authors proposed a hypothesis of “system hysteresis” as the basic mechanism of minocycline’s extended efficacy against intracellular *Mtb* and termed it as “pharmacologic memory” allowing intermittent dosing of minocycline. The authors found that in the HFS-TB, the minocycline-containing once-a-week combination regimen was as effective as daily therapy. Clinical validation of these findings is warranted.

While the development of new drugs and combination regimens is needed to further shorten both drug-susceptible and MDR-TB, identifying new methods to determine the drug efficacy is equally important to expedite the process. The study by Mockeliunas et al. describes a pharmacometric model-based approach that could be used in the clinical trials designed to determine the early bactericidal activity of the drugs. The authors used an estimate of MGIT-generated TTP slope, variability in TTP slope, impact of covariates, and drug PKs to link all these covariates with efficacy. The seven-step approach of this paper that employed the expertise of microbiologists, clinicians, and pharmacometricians could potentially be used to evaluate the EBA of monotherapies as well as combination regimens.

The culture of *Mtb* takes many weeks to grow. Therefore, alternative methods and biomarkers that can be used to measure the changes in the bacterial burden upon drug treatment are needed. The study by Alsoud et al. describes the role of TTP and CFU to develop a biomarker for TB drug development and to establish the efficacy of drugs in EBA studies. To develop a quantitative biomarker model for TTP and CFU to inform future EBA studies, paired TTP and CFU data from 83 patients with uncomplicated pulmonary TB receiving rifampicin monotherapy (dose range 10–40 mg/kg) was included. These patients were from the HIGHRIF1 study (Boeree et al., 2015). While the proposed PK-CFU-TTP model was able to identify the relationship between CFU and TTP, the data on the bacterial growth phase, and the difference in the growth rate of different *Mtb* metabolic subpopulations including nonreplicating persisters was not estimated. Further, the authors caution against extrapolation of their findings outside the range (10–40 mg/kg) as well as consideration of bacterial susceptibly and treatment duration when using such a modeling approach.

Finally, the study by Wasmann et al. addresses the treatment approach in special populations. This study describes the methods to develop a new dosing strategy to facilitate short-course regimens for TB meningitis in children, based on globally available new drug formulations. The authors simulated several dosing options in a virtual population of children using population PK modeling. We agree with the authors that the development of dosing strategies should be informed by efficacy, safety, and PK data. Similarly, the availability of age-appropriate drug formulations is important for program implementation considerations. Given the age and size, with a given mg/kg dose, the drug exposure predictions in children are more complex compared to adults. Therefore, it is important to consider developmental PK that can otherwise result in toxic or subtherapeutic concentrations. The authors also provided a simple MS Excel dosing tool to allow clinicians to develop the dosing strategy for TB meningitis in situations where a recommended formulation is not available.
